# Acute viral respiratory infections in children before and during the COVID-19 pandemic in a Peruvian reference hospital

**DOI:** 10.17843/rpmesp.2026.433.15564

**Published:** 2026-09-04

**Authors:** Jhonatan R. Mejia, Daniel Fernandez-Guzman, Medalit Luna-Vilchez, Alvaro Taype-Rondan

**Affiliations:** 1 Carrera de Medicina Humana, Universidad Científica del Sur, Lima, Peru. Universidad Científica del Sur Carrera de Medicina Humana Universidad Científica del Sur Lima Peru; 2 Unidad de Atención Integral Especializada, Instituto Nacional de Salud del Niño San Borja, Lima, Peru. Unidad de Atención Integral Especializada Instituto Nacional de Salud del Niño San Borja Lima Peru; 3 EviSalud - Evidencias en Salud, Lima, Peru. EviSalud Evidencias en Salud Lima Peru; 4 Unidad de Investigación para la Generación y Síntesis de Evidencias en Salud, Universidad San Ignacio de Loyola, Lima, Peru. Universidad San Ignacio de Loyola Unidad de Investigación para la Generación y Síntesis de Evidencias en Salud Universidad San Ignacio de Loyola Lima Peru

**Keywords:** Pediatrics, Respiratory Infections, Epidemiology, Viruses, Peru

## Abstract

Our objective was to evaluate the frequency and characteristics of acute respiratory infections (ARI) with confirmed viral etiology in children treated at a Peruvian referral hospital before and during the COVID-19 pandemic between 2019 and 2022. We conducted a descriptive study based on the detection records of adenovirus, influenza A and B (IA and IB), parainfluenza 1-3 (PIV), metapneumovirus (hMPV), and respiratory syncytial virus (RSV). Of 1740 episodes (1220 patients), 14.4% tested positive: 196 (78.0%) RSV, 23 (9.2%) IA, 14 (5.6%) PIV-3, and 13 (5.2%) hMPV. In children under five years of age, RSV predominated, while in children over five years of age, it was IA. The frequency of ARI was lower with increasing age, during the pandemic (2020-2021), and the spring and summer seasons. The higher frequency of respiratory viruses in 2022 highlights the need to maintain and strengthen surveillance and preparedness against these outbreaks.

## INTRODUCTION

Acute respiratory infections (ARI), often of viral origin, constitute one of the main causes of morbidity and mortality worldwide, especially in children, older adults, and immunosuppressed individuals ^1^. In 2016, respiratory syncytial virus (RSV) and influenza caused 17 and 9.1 episodes of lower respiratory tract infection per 1000 children under five years of age worldwide, with mortality rates of 6.5 and 1.3 per 100,000, respectively ^2^.

The COVID-19 pandemic led to the implementation of public health measures such as lockdown, social distancing, mask use, and school closures. These measures, aimed at curbing the spread of SARS-CoV-2, also modified the transmission dynamics of other respiratory viruses (RV) ^3^. In China, a report indicated a reduction in hospitalizations for ARI during this period, probably due to lower viral circulation ^4^. However, in limited-resource settings like Peru, evidence remains scarce.

In Peru, ARIs continue to be a major cause of pediatric hospitalization. Although the Government implemented several measures during the COVID-19 pandemic ^5^, the frequency of ARIs before and during this period has not been studied in depth. The Instituto Nacional de Salud del Niño-San Borja (INSN-SB), in Lima, is one of the main pediatric hospitals in Peru and one of the few that routinely evaluates the etiology of ARIs. Between 2017 and 2018, it was reported that 13.4% of the analyzed patients from INSN-SB tested positive for RV, mainly RSV (10.7%), with variations according to season and age group ^6^.

Understanding the frequency and viral etiology of ARIs in Peru is essential for designing prevention and treatment strategies. Likewise, analyzing trends during the pandemic can provide relevant information for the formulation of public health policies in the post-pandemic stage. Therefore, the objective of this study was to estimate the frequency and characteristics of ARIs with confirmed viral etiology in children treated at INSN-SB before and during the COVID-19 pandemic.

**KEY MESSAGES**

Motivation for study. Although there are studies on acute respiratory infections, the circulation of respiratory viruses in children in Peru before and during the COVID-19 pandemic is unknown.

Main findings. One in ten episodes had a confirmed viral etiology in a reference hospital in Peru. Of these, respiratory syncytial virus predominated in children under five years old and influenza A in those over five years old. The frequency decreased during the pandemic (2020-2021) and increased in 2022.

Public health implications. The higher frequency in 2022 highlights the need to maintain and strengthen surveillance and preparedness against future outbreaks of respiratory viruses.

## THE STUDY

A descriptive study was conducted based on records of ARI episodes of patients treated in the different services of INSN-SB. Retrospective data were collected from episodes diagnosed by direct immunofluorescence (DIF) in samples analyzed between January 2019 and August 2022. Access to the database was granted on April 25, 2023.

Episodes were included in patients aged 0 to 18 years with ARI symptoms, hospitalized or ambulatory (outpatient and emergency), with an adequate nasopharyngeal swab sample. Episodes from patients external to the institution or with an inadequate sample were excluded. ARIs were defined as the presence of one or more of the following symptoms during a period of less than 14 days: cough, rhinorrhea, nasal obstruction, odynophagia, otalgia, wheezing, or respiratory distress, with or without fever ^7^.

The DIF test was performed in patients diagnosed with IRA. Between 2019 and 2021, recruitment was carried out by clinical assistants and infectology staff; however, this process was not protocolized and depended on clinical criteria. Additionally, there was no systematic flow that covered all services nor personnel exclusively assigned to sample collection for infections other than COVID-19. During the first two years of the pandemic, activities aimed at identifying IRA cases decreased due to the public health measures implemented against COVID-19. Starting in 2022, a protocolized RV surveillance was established, with personnel assigned daily to case identification and systematic sample collection in all services.

Samples were collected and processed by the Department of Clinical Pathology ^8^. Nasopharyngeal samples were obtained using sterile swabs, placed in viral transport media, and maintained between 2 and 8 °C until processing. For this, personnel followed the protocol of the commercial kit *D3 Ultra 8™ DFA Respiratory Virus Screening & Identification* for the detection of adenovirus (ADV), influenza A and B (IA and IB), parainfluenza 1-3 (PIV), metapneumovirus (hMPV), and RSV ^9^.

A trained team performed the reading with the ZEISS Axio Scope.A1 fluorescence microscope. A suitable sample was considered when it presented at least 20 columnar epithelial cells and a positive result for RV when two or more cells with apple-green intracellular fluorescence were observed.

Clinical data were obtained from epidemiological records and electronic medical records, and were independently digitized by two researchers in Microsoft Excel 2016 (Microsoft Corporation, WA, USA) for quality control. This methodology was previously reported ^6^.

Information was collected on age during the ARI episode, sex, sampling year, type of care (outpatient, emergency, or hospitalization), season of the year (autumn: March 19 to June 20, winter: June 20 to September 22, spring: September 22 to December 21, summer: December 21 to March 19) ^10^, period in relation to the COVID-19 pandemic (pre-pandemic, first, second, third, and fourth wave) according to the Ministry of Health of Peru ^11^, type of RV and viral coinfections.

The analysis was performed in Stata*®* v.17.0 (StataCorp, TX, USA). Age was summarized as median and interquartile range (IQR), categorical variables as absolute and relative frequencies for both ARI episodes (n=1740) and panels with positive RV results (n=250). Additionally, the frequency of ARI episodes, RV positivity, and their distribution between 2019, 2020-2021, and 2022 were described.

The study was approved by the Research Ethics Committee of the INSN-SB (INSN PI714 CONSTANCIA 015-2023). The confidentiality of the analyzed records was maintained.

## FINDINGS

1740 episodes of ARI were analyzed in 1220 patients (1.4 episodes/patient) evaluated with DIF for n co viral etiology. In total, 250 (14.4%) panels were positive for any RV, with variations by year: 19.5% in 2019, 3.4% in 2020, 8.5% in 2021, and 21.9% in 2022 (Table 1, Figure 1A). Furthermore, a marked reduction in episodes was observed in 2020 (13.3%), followed by a progressive increase in 2021 (34.7%) and 2022 (34.3%) (Figure 1A).

**Table 1:** Frequency of acute viral respiratory infections at the Instituto Nacional de Salud del Niño-San Borja, January 2019 to August 2022.

Year	Episodes of ARI	Confirmed episodes of respiratory viruses							
		RV (+)	RSV^b^	AI^b^	PIV-3^b^	hMPV^b^	PIV-1^b^	IB^b^	PIV-2^b^
	n (%)	n (%)	n (%)	n (%)	n (%)	n (%)	n (%)	n (%)	n (%)
2019	308 (17.7)	60 (19.5)	40 (66.7)	12 (20.0)	2 (3.3)	3 (5.0)	1 (1.7)	2 (3.3)	0
2020	232 (13.3)	8 (3.4)	6 (75.0)	0 (0.0)	0 (0.0)	1 (12.5)	1 (12.5)	0 (0.0)	0 (0.0)
2021	603 (34.7)	51 (8.5)	48 (94.1)	0 (0.0)	1 (2.0)	3 (5.9)	0 (0.0)	0 (0.0)	0 (0.0)
2022^c^	597 (34.3)	131 (21.9)	102 (77.9)	11 (8.4)	11 (8.4)	6 (4.6)	1 (0.8)	0 (0.0)	0 (0.0)
Total	1740 (100.0)	250 (14.4)	196 (78.0)	23 (9.2)	14 (5.6)	13 (5.2)	3 (1.2)	2 (0.8)	0 (0.0)

ARI: acute respiratory infection; RV: respiratory virus; RSV: respiratory syncytial virus; IA: influenza A; PIV: parainfluenza virus; hMPV: metapneumovirus; IB: influenza B.

a The percentages correspond to the total number of acute respiratory infections (N=1740).

b The percentages correspond to the total number of confirmed respiratory virus episodes for the evaluated period.

c Until August 2022.

**Figure 1:**
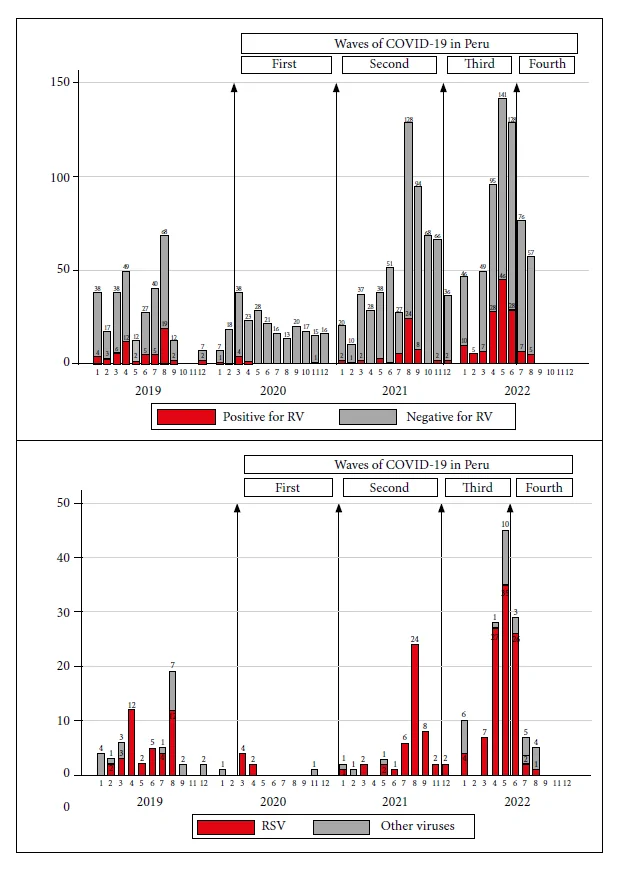
A) Frequency of episodes of acute respiratory infections (ARI) confirmed by direct immunofluorescence. B) Frequency of episodes of ARI of viral etiology confirmed by direct immunofluorescence.

RV: respiratory virus, RSV: respiratory syncytial virus.

Most tests (28.1%) corresponded to children younger than one year. Viral positivity was lower in the 5 to 10-year-old (8.6%) and older than 10-year-old (9.2%) groups (table 2, figure 2). The highest detection was recorded in 2022 (21.9%), during the third wave of COVID-19 (24.5%) and in autumn (20%). Positivity was lower in 2020 (3.4%), 2021 (8.5%), as well as in spring (3.7%) and summer (9.7%).

**Table 2:** Characteristics of ARI episodes with a positive result for respiratory viruses at the Instituto Nacional de Salud del Niño-San Borja, January 2019 to August 2022.

Characteristics		Episodes of ARI (n=1740)	RV negative (n=1490)	RV positive (n=250)
		n (%)	n (%)	n (%)
Age in months, median (IQR)		26 (10‒70)	29 (11‒74)	20 (8‒41)
Age group				
	< 1 year	489 (28.1)	400 (81.8)	89 (18.2)
	1 to < 2 years	334 (19.2)	281 (84.1)	53 (15.9)
	2 to < 5 years	420 (24.1)	356 (84.8)	64 (15.2)
	5 years to 10 years	279 (16.0)	255 (91.4)	24 (8,6)
	10 years	218 (12.6)	198 (90.8)	20 (9.2)
Sex				
	Female	765 (44.0)	647 (84.6)	118 (15.4)
	Male	975 (56.0)	843 (86.5)	132 (13.5)
Type of care				
	Hospitalization	1599 (91.9)	1371 (85.7)	228 (14.3)
	Outpatient clinic	45 (2.6)	40 (88.9)	5 (11.1)
	Emergency	96 (5.5)	79 (82.3)	17 (17.7)
Year				
	2019	308 (17.7)	248 (80.5)	60 (19.5)
	2020	232 (13.3)	224 (96.6)	8 (3.4)
	2021	603 (34.7)	552 (91.5)	51 (8.5)
	2022 (until August)	597 (34.3)	466 (78.1)	131 (21.9)
Season of the year				
	Autumn	640 (36.8)	512 (80.0)	128 (20.0)
	Winter	589 (33.8)	502 (85.2)	87 (14.8)
	Spring	242 (13.9)	233 (96.3)	9 (3.7)
	Summer	269 (15.5)	243 (90.3)	26 (9.7)

ARI: acute respiratory infection; RV: respiratory virus; IQR: interquartile range.

**Figure 2:**
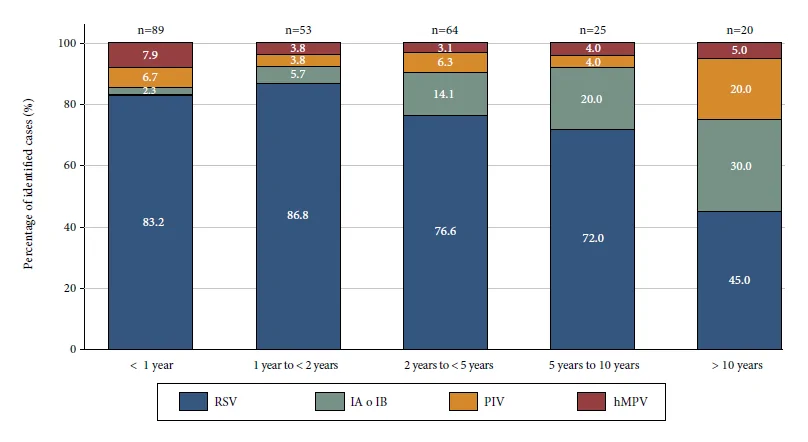
Etiology of acute viral respiratory infection episodes confirmed by direct immunofluorescence, by age group.

RSV: respiratory syncytial virus; IA or IB: influenza A or influenza B; PIV: parainfluenza virus; hMPV: human metapneumovirus.

Among positive cases, RSV was the most frequent (78.0%), but it had a decrease in 2020 (2.4%) (figure 1B). In contrast, in 2022, the detection of other viruses increased: IA (4.4%), PIV 3 (4.4%) and hMPV (2.4%) (figure 1B, supplementary table 1). RSV predominated in children under one year (83.2%) and from 1 to 2 years (86.8%), with peaks in 2021 (94.1%), the second wave of COVID-19 (93.9%) and in autumn (88.3%). IA and IB represented 10.0% of cases, with higher frequency in children older than 10 years (30.0%), especially in 2019 (23.3%) and in summer (38.5%). PIV (6.8%) was detected mainly during the fourth wave (43.8%). hMPV (5.2%) was more frequent in spring (33.3%) (supplementary table 1). Annual frequencies are presented in supplementary tables 2 and 3.

## DISCUSSION

The trends of acute viral respiratory infection observed in this study show a reduction in RV positivity during the first and second waves of COVID-19 in Peru (2020-2021), followed by a subsequent increase. Similarly, the frequency of RV decreased during the pandemic in China ^4^ and in Latin America during the winter and autumn of 2020 (seasons with pre-pandemic outbreaks) ^12^. Furthermore, this pattern was consistent with a study focused specifically on RSV conducted from a pediatric registry of 260 facilities in Tokyo, which did not report IRA outbreaks due to RSV in 2020, however, the authors observed a high frequency in 2021 ^13^. This was probably due to public health measures such as lockdown and social distancing, which limited viral transmission and child socialization ^14^.

In Peru, a strict national quarantine likely contributed to more than 100 days of reduced RV circulation ^15^. Nevertheless, reduced attention to mild pathologies in different health centers could lead to underreporting. On the other hand, the high frequency of RV in 2021-2022 suggests a possible rebound effect, underscoring the importance of maintaining and strengthening epidemiological surveillance of RV.

The lowest frequency of ARI occurred in older age groups (5-10 years and >10 years), the years 2020 and 2021, and the spring and summer seasons. These findings coincide with studies from China ^(^^4^, and Germany ^16^^)^ which showed a lower incidence of ARI in older children and during the early phase of the pandemic. It is important to note that no cases of IA or IB were registered in 2020-2021, with a rebound in 2022, a trend also observed in India ^17^.

Seasonal patterns coincided with findings from South Africa ^18^ and Argentina ^19^. In Peru, a study found that RSV reached its circulation peaks during autumn and winter of 2017 and 2018 ^6^, which agrees with our results. The resurgence of cases in 2021 and 2022 can be attributed, in part, to the strengthening of epidemiological surveillance.

Non-pharmacological interventions, such as mask use, hand hygiene, social distancing, and quarantine, probably reduced RV transmission during the pandemic ^14^. While these measures were beneficial, the interruption of viral circulation in 2020 may have consequences since usually 90% of children experience at least one viral ARI in the first two years of life ^20^, which contributes to the development of their immunity. Lower exposure during the pandemic could have left children more susceptible to future infections, which could translate into greater respiratory morbidity and mortality in the coming years. These findings highlight the need to improve diagnostic and preventive strategies by strengthening epidemiological surveillance in health facilities. The increase in RV cases in 2022 suggests the possibility of future outbreaks, emphasizing the importance of preparedness and surveillance in Peru for similar future scenarios.

Among the limitations, it should be mentioned that this study was conducted in a reference hospital, which may restrict the generalizability of the findings to other levels of care. Furthermore, cases attended at other care centers and episodes that did not seek care might have been omitted. Additionally, the lower frequency of ARI in 2020 may be partially explained by reduced attendance at health services due to fear of COVID-19. Likewise, differences in the capture strategy between 2019-2021 (non-standardized) and 2022 (standardized active viral surveillance) might have influenced the frequency of episodes and observed viral positivity, limiting comparability between periods, although RV frequencies during 2019 remained similar to 2022. On the other hand, the 2022 data correspond only up to August, which might limit comparison with previous years; however, a viral positivity higher than that recorded during the first years of the pandemic was already observed. Finally, causality cannot be established due to the study design. However, the study presents the strength of having focused on virologically confirmed ARI cases through DIF tests. Furthermore, we provide detailed analyses by virus type, age group, season, and year, covering trends until August 2022.

In conclusion, RSV was the main viral cause of ARI, especially in children under five years old, while influenza predominated in older age groups treated at INSN-SB. The frequency of ARI was lower with increasing age, during the first two years of the COVID-19 pandemic (2020 and 2021), and during the spring and summer seasons. The higher frequency of RV observed in 2022 suggests the need to maintain and strengthen surveillance and outbreak preparedness in Peru to face future public health challenges related to respiratory infections.
