# Supplementary material for: Acute viral respiratory infections in children before and during the COVID-19 pandemic in a Peruvian reference hospital
Source: Rev Peru Med Exp Salud Publica. 2026 Sep 4;43(3):349–55. doi: 10.17843/rpmesp.2026.433.15564 (PMC13718560; doi:10.17843/rpmesp.2026.433.15564)
Supplement: Supplementary file 2 [file rpmesp-43-03-15564-s001.docx]

**Material suplementario**

**Tabla S1. Características por etiología viral de los episodios de IRA atendidos en el Instituto Nacional de Salud del Niño San Borja 2019-2022. (N=250)**

| **Características** | **VSR**  **(n=195)** | **IA o IB (n=25)** | **PIV (1-3) (n=17)** | **hMPV (n=13)** |
| --- | --- | --- | --- | --- |
|  | **Positivo** | **Positivo** | **Positivo** | **Positivo** |
|  | **n (%)** | **n (%)** | **n (%)** | **n (%)** |
| **Grupo etario** |  |  |  |  |
| < 1 año | 74 (83,2) | 2 (2,3) | 6 (6,7) | 7 (7,9) |
| 1 año a < 2 años | 46 (86,8) | 3 (5,7) | 2 (3,8) | 2 (3,8) |
| 2 años a 5 años | 49 (76,6) | 9 (14,1) | 4 (6,3) | 2 (3,1) |
| 5 años a 10 años | 18 (75,0) | 5 (20,8) | 1 (4,2) | 1 (4,2) |
| >10 años | 9 (45,0) | 6 (30,0) | 4 (20,0) | 1 (5,0) |
| **Sexo** |  |  |  |  |
| Femenino | 95 (80,5) | 12 (10,2) | 8 (6,8) | 4 (3,4) |
| Masculino | 101 (76,5) | 13 (9,9) | 9 (6,8) | 9 (6,8) |
| **Tipo de atención** |  |  |  |  |
| Consultorio externo | 1 (20,0) | 4 (80,0) | 0 (0,0) | 0 (0,0) |
| Emergencia | 13 (76,5) | 3 (17,7) | 0 (0,0) | 1 (5,9) |
| Hospitalización | 182 (79,8) | 18 (7,9) | 17 (7,5) | 12 (5,3) |
| **Año** |  |  |  |  |
| 2019 | 40 (66,7) | 14 (23,3) | 3 (5,0) | 3 (5,0) |
| 2020 | 6 (75,0) | 0 (0,0) | 1 (12,5) | 1 (12,5) |
| 2021 | 48 (94,1) | 0 (0,0) | 1 (2,0) | 3 (5,9) |
| 2022 | 102 (77,9) | 11 (8,4) | 12 (9,2) | 6 (4,6) |
| **Ola de COVID-19** |  |  |  |  |
| No | 40 (65,6) | 14 (23,0) | 4 (6,6) | 3 (4,9) |
| Primera | 7 (87,5) | 0 (0,0) | 0 (0,0) | 1 (12,5) |
| Segunda | 46 (93,9) | 0 (0,0) | 1 (2,0) | 3 (6,1) |
| Tercera | 97 (83,6) | 10 (8,6) | 5 (4,3) | 4 (3,5) |
| Cuarta | 6 (37,5) | 1 (6,3) | 7 (43,8) | 2 (12,5) |
| **Estación del año** |  |  |  |  |
| Otoño | 113 (88,3) | 6 (4,7) | 5 (3,9) | 4 (3,1) |
| Invierno | 66 (75,9) | 9 (10,3) | 9 (10,3) | 4 (4,6) |
| Primavera | 6 (66,7) | 0 (0,0) | 0 (0,0) | 3 (33,3) |
| Verano | 11 (42,3) | 10 (38,5) | 3 (11,5) | 2 (7,7) |

IRA: Infección respiratoria aguda; VSR: virus sincitial respiratorio; IA: Influenza A; PIV: Parainfluenza virus; hMPV: Metapneumovirus; IB: Influenza B.
**Tabla S2. Características de los episodios de IRA por año de atención en el Instituto Nacional de Salud del Niño San Borja 2019-2022. (N=1740)**

| **Características** | **2019  (n=308)** | **2020 (n=232)** | **2021 (n=603)** | **2022 (n=597)** |
| --- | --- | --- | --- | --- |
|  | **n (%)** | **n (%)** | **n (%)** | **n (%)** |
| **Grupo etario** |  |  |  |  |
| < 1 año | 111 (36,0) | 80 (34,5) | 149 (24,7) | 149 (25,0) |
| 1 año a < 2 años | 62 (20,1) | 44 (19,0) | 120 (19,9) | 108 (18,1) |
| 2 años a 5 años | 62 (20,1) | 44 (19,0) | 148 (24,5) | 166 (27,8) |
| 5 años a 10 años | 36 (11,7) | 36 (15,5) | 105 (17,4) | 102 (17,1) |
| >10 años | 37 (12,0) | 28 (12,1) | 81 (13,4) | 72 (12,1) |
| **Sexo** |  |  |  |  |
| Femenino | 157 (51,0) | 83 (35,8) | 253 (42,0) | 272 (45,6) |
| Masculino | 151 (49,0) | 149 (64,2) | 350 (58,0) | 325 (54,4) |
| **Tipo de atención** |  |  |  |  |
| Consultorio externo | 284 (92,2) | 227 (97,8) | 562 (93,2) | 526 (88,1) |
| Emergencia | 22 (7,1) | 5 (2,2) | 11 (1,8) | 7 (1,2) |
| Hospitalización | 2 (0,7) | 0 (0,0) | 30 (5,0) | 64 (10,7) |
| **Positividad de VR** |  |  |  |  |
| No | 248 (80,5) | 224 (96,6) | 552 (91,5) | 466 (78,1) |
| Sí | 60 (19,5) | 8 (3,5) | 51 (8,5) | 131 (21,9) |
| **Ola de COVID-19** |  |  |  |  |
| No | 308 (100,0) | 28 (12,1) | 0 (0,0) | 0 (0,0) |
| Primera | 0 (0,0) | 204 (87,9) | 8 (1,3) | 0 (0,0) |
| Segunda | 0 (0,0) | 0 (0,0) | 574 (95,2) | 0 (0,0) |
| Tercera | 0 (0,0) | 0 (0,0) | 21 (3,5) | 453 (75,9) |
| Cuarta | 0 (0,0) | 0 (0,0) | 0 (0,0) | 144 (24,1) |
| **Estación del año** |  |  |  |  |
| Otoño | 94 (30,5) | 87 (37,5) | 110 (18,2) | 349 (58,5) |
| Invierno | 125 (40,6) | 46 (19,8) | 245 (40,6) | 173 (29,0) |
| Primavera | 8 (2,6) | 50 (21,6) | 184 (30,5) | 0 (0,0) |
| Verano | 81 (26,3) | 49 (21,1) | 64 (10,6) | 75 (12,6) |

IRA: Infección respiratoria aguda; VR: Virus respiratorio

**Tabla S3. Características de las IRA de etiología viral por año de atención en el Instituto Nacional de Salud del Niño San Borja. 2019-2022 (N=250)**

| **Características** | **2019  (n=60)** | **2020 (n=8)** | **2021 (n=51)** | **2022 (n=131)** |
| --- | --- | --- | --- | --- |
|  | **n (%)** | **n (%)** | **n (%)** | **n (%)** |
| **Grupo etario** |  |  |  |  |
| < 1 año | 30 (50,0) | 2 (25,0) | 18 (35,3) | 39 (29,8) |
| 1 año a < 2 años | 14 (23,3) | 3 (37,5) | 8 (15,7) | 28 (21,4) |
| 2 años a 5 años | 9 (15,0) | 2 (25,0) | 13 (25,5) | 40 (30,5) |
| 5 años a 10 años | 2 (3,3) | 0 (0,0) | 9 (17,7) | 13 (9,9) |
| >10 años | 5 (8,3) | 1 (12,5) | 3 (5,9) | 11 (8,4) |
| **Sexo** |  |  |  |  |
| Femenino | 29 (48,3) | 0 (0,0) | 26 (51,0) | 63 (48,1) |
| Masculino | 31 (51,7) | 8 (100,0) | 25 (49,0) | 68 (51,9) |
| **Tipo de atención** |  |  |  |  |
| Consultorio externo | 54 (90,0) | 8 (100,0) | 47 (92,2) | 119 (90,8) |
| Emergencia | 4 (6,7) | 0 (0,0) | 1 (2,0) | 0 (0,0) |
| Hospitalización | 2 (3,3) | 0 (0,0) | 3 (5,9) | 12 (9,2) |
| **Ola de COVID-19** |  |  |  |  |
| No | 60 (100,0) | 1 (12,5) | 0 (0,0) | 0 (0,0) |
| Primera | 0 (0,0) | 7 (87,5) | 1 (2,0) | 0 (0,0) |
| Segunda | 0 (0,0) | 0 (0,0) | 49 (96,1) | 0 (0,0) |
| Tercera | 0 (0,0) | 0 (0,0) | 1 (2,0) | 115 (87,8) |
| Cuarta | 0 (0,0) | 0 (0,0) | 0 (0,0) | 16 (12,2) |
| **Estación del año** |  |  |  |  |
| Otoño | 22 (36,7) | 5 (62,5) | 5 (9,8) | 96 (73,3) |
| Invierno | 28 (46,7) | 0 (0,0) | 37 (72,6) | 22 (16,8) |
| Primavera | 2 (3,3) | 1 (12,5) | 6 (11,8) | 0 (0,0) |
| Verano | 8 (13,3) | 2 (25,0) | 3 (5,9) | 13 (9,9) |

IRA: Infección respiratoria aguda
