# Supplementary material for: Acute viral respiratory infections in children before and during the COVID-19 pandemic in a Peruvian reference hospital
Source: Rev Peru Med Exp Salud Publica. 2026 Sep 4;43(3):349–55. doi: 10.17843/rpmesp.2026.433.15564 (PMC13718560; doi:10.17843/rpmesp.2026.433.15564)
Supplement: Translation

# Translation: Infecciones respiratorias virales agudas en niños antes y durante la pandemia de COVID-19 en un hospital de referencia peruano

**Authors:** Jhonatan R. Mejia, Daniel Fernandez-Guzman, Medalit Luna-Vilchez, Alvaro Taype-Rondan

**Affiliations:** 1 Universidad Científica del Sur, Carrera de Medicina Humana, Lima, Perú.; 2 Unidad de Atención Integral Especializada, Instituto Nacional de Salud del Niño San Borja, Lima, Perú.; 3 EviSalud - Evidencias en Salud, Lima, Perú.; 4 Universidad San Ignacio de Loyola, Unidad de Investigación para la Generación y Síntesis de Evidencias en Salud, Lima, Perú.

**Keywords:** Niños, Infecciones del Sistema Respiratorio, Epidemiología, Virus, Perú

## Abstract

Nuestro objetivo fue evaluar la frecuencia y características de infecciones respiratorias agudas (IRA) con etiología viral confirmada en niños atendidos en un hospital de referencia peruano antes y durante la pandemia de COVID-19 entre 2019 y 2022. Realizamos un estudio descriptivo basado en los registros de detección de adenovirus, influenza A y B (IA y IB), parainfluenza 1-3 (PIV), metapneumovirus (hMPV) y virus sincitial respiratorio (VSR). De 1740 episodios (1220 pacientes), 14,4% resultaron positivos: 196 (78,0%) VSR, 23 (9,2%) IA, 14 (5,6%) PIV-3 y 13 (5,2%) hMPV. En menores de cinco años predominó el VSR, mientras que en mayores de cinco años fue la IA. La frecuencia de IRA fue menor a mayor edad, durante la pandemia (2020-2021) y las estaciones de primavera y verano. La mayor frecuencia de virus respiratorios en 2022 resalta la necesidad de mantener y reforzar la vigilancia y preparación frente a estos brotes.

## INTRODUCCIÓN

Las infecciones respiratorias agudas (IRA), a menudo de origen viral, constituyen una de las principales causas de morbilidad y mortalidad a nivel mundial, en especial en niños, adultos mayores e individuos inmunosuprimidos ^1^. En 2016, el virus sincitial respiratorio (VSR) y la influenza ocasionaron 17 y 9,1 episodios de infección respiratoria baja por cada 1000 niños menores de cinco años en el mundo, con tasas de mortalidad de 6,5 y 1,3 por cada 100 000, respectivamente ^2^.

La pandemia de COVID-19 condujo a la implementación de medidas de salud pública como el confinamiento, el distanciamiento social, el uso de mascarillas y el cierre de escuelas. Estas medidas, orientadas a frenar la propagación del SARS-CoV-2, también modificaron la dinámica de transmisión de otros virus respiratorios (VR) ^3^. En China, un reporte indicó una reducción en las hospitalizaciones por IRA durante este periodo, probablemente debido a la menor circulación viral ^4^. Sin embargo, en escenarios de recursos limitados como el Perú, la evidencia sigue siendo escasa.

En el Perú, las IRA continúan siendo una causa importante de hospitalización pediátrica. Si bien, el Gobierno aplicó varias medidas durante la pandemia de COVID-19 ^5^, la frecuencia de las IRA antes y durante este periodo no ha sido estudiada en profundidad. El Instituto Nacional de Salud del Niño-San Borja (INSN-SB), en Lima, es uno de los principales hospitales pediátricos del Perú y uno de los pocos que evalúa rutinariamente la etiología de las IRA. Entre 2017 y 2018, se reportó que el 13,4 % de los pacientes analizados del INSN-SB resultaron positivos para VR, principalmente VSR (10,7%), con variaciones según la estación y el grupo etario ^6^.

Comprender la frecuencia y etiología viral de las IRA en el Perú es fundamental para diseñar estrategias de prevención y tratamiento. Asimismo, analizar las tendencias durante la pandemia puede aportar información relevante para la formulación de políticas de salud pública en la etapa pospandemia. Por ello, el objetivo de este estudio fue estimar la frecuencia y características de las IRA con etiología viral confirmada en niños atendidos en el INSN-SB antes y durante la pandemia de COVID-19.

**MENSAJES CLAVE**

Motivación para realizar el estudio. Aunque existen estudios sobre infecciones respiratorias agudas, se desconoce la circulación de virus respiratorios en niños en el Perú antes y durante la pandemia de COVID-19.

Principales hallazgos. Uno de cada diez episodios tuvo una etiología viral confirmada en un hospital de referencia del Perú. De ellos, predominó el virus sincitial respiratorio en menores de cinco años e influenza A en mayores de cinco años. La frecuencia disminuyó durante la pandemia (2020-2021) y aumentó en 2022.

Implicancias en salud pública. La mayor frecuencia en 2022 resalta la necesidad de mantener y reforzar la vigilancia y preparación frente a futuros brotes de virus respiratorios.

## EL ESTUDIO

Se realizó un estudio descriptivo con base en los registros de episodios de IRA de pacientes atendidos en los diferentes servicios del INSN-SB. Se recopilaron datos retrospectivos de episodios diagnosticados mediante inmunofluorescencia directa (IFD) en muestras analizadas entre enero de 2019 y agosto de 2022. El acceso a la base de datos se otorgó el 25 de abril de 2023.

Se incluyeron episodios en pacientes de 0 a 18 años con sintomatología de IRA, hospitalizados o ambulatorios (consulta externa y emergencia), con muestra de hisopado nasofaríngeo adecuada. Se excluyeron episodios de pacientes externos a la institución o con muestra inadecuada. Las IRA se definieron como la presencia de uno o más de los siguientes síntomas durante un periodo menor de 14 días: tos, rinorrea, obstrucción nasal, odinofagia, otalgia, sibilancias o dificultad respiratoria, con o sin fiebre ^7^.

Se realizó la prueba de IFD en los pacientes diagnosticados con IRA. Entre 2019 y 2021, el reclutamiento estuvo a cargo de asistentes clínicos y personal de infectología; sin embargo, este proceso no estuvo protocolizado y dependía del criterio clínico. Además, no existía un flujo sistemático que abarcara todos los servicios ni personal asignado exclusivamente a la toma de muestras para infecciones distintas de la COVID-19. Durante los dos primeros años de la pandemia, las actividades destinadas a identificar casos de IRA disminuyeron debido a las medidas de salud pública implementadas frente a la COVID-19. A partir de 2022, se estableció una vigilancia protocolizada de VR, con personal asignado diariamente a la identificación de casos y la toma sistemática de muestras en todos los servicios.

Las muestras fueron recolectadas y procesadas por el Departamento de Patología Clínica ^8^. Las muestras nasofaríngeas se obtuvieron mediante hisopos estériles, se colocaron en medios de transporte viral y se mantuvieron entre 2 y 8 °C hasta su procesamiento. Para ello, el personal siguió el protocolo del kit comercial *D3 Ultra 8™ DFA Respiratory Virus Screening & Identification* para la detección de adenovirus (ADV), influenza A y B (IA y IB), parainfluenza 1-3 (PIV), metapneumovirus (hMPV) y VSR ^9^.

Un equipo entrenado realizó la lectura con el microscopio de fluorescencia ZEISS Axio Scope.A1. Se consideró una muestra adecuada cuando presentó al menos 20 células epiteliales columnares y un resultado positivo para VR cuando se observaron dos o más células con fluorescencia intracelular verde manzana.

Los datos clínicos se obtuvieron de los registros epidemiológicos e historias clínicas electrónicas, y fueron digitalizados de manera independiente por dos investigadores en Microsoft Excel 2016 (Microsoft Corporation, WA, EE.UU.) para el control de calidad. Esta metodología fue reportada previamente ^6^.

Se recolectó información sobre la edad durante el episodio de IRA, sexo, año de muestreo, tipo de atención (consulta externa, emergencia u hospitalización), estación del año (otoño: 19 de marzo al 20 de junio, invierno: 20 de junio al 22 de septiembre, primavera: 22 de septiembre al 21 de diciembre, verano: 21 de diciembre al 19 de marzo) ^10^, periodo en relación a la pandemia de COVID-19 (prepandemia, primera, segunda, tercera y cuarta ola) de acuerdo con el Ministerio de Salud del Perú ^11^, tipo de VR y coinfecciones virales.

El análisis fue realizado en Stata*®* v.17.0 (StataCorp, TX, EE.UU.). Se resumió la edad como mediana y rango intercuartílico (RIC), las variables categóricas como frecuencias absolutas y relativas tanto para los episodios de IRA (n=1740) y los paneles con resultados VR positivos (n=250). Adicionalmente, se describió la frecuencia de episodios de IRA, la positividad a VR y su distribución entre 2019, 2020-2021 y 2022.

El estudio fue aprobado por el Comité de Ética de Investigación del INSN-SB (INSN PI714 CONSTANCIA 015-2023). Se mantuvo la confidencialidad de los registros analizados.

## HALLAZGOS

Se analizaron 1740 episodios de IRA en 1220 pacientes (1,4 episodios/paciente) evaluados con IFD para n co etiología viral. En total, 250 (14,4%) paneles fueron positivos para algún VR, con variaciones según año: 19,5% en 2019, 3,4% en 2020, 8,5% en 2021 y 21,9% en 2022 (tabla 1, figura 1A). Además, se observó una marcada reducción de episodios en 2020 (13,3%), seguida de un incremento progresivo en 2021 (34,7%) y 2022 (34,3%) (figura 1A).

**Tabla 1:** Frecuencia de infecciones respiratorias virales agudas en el Instituto Nacional de Salud del Niño-San Borja, enero 2019 a agosto 2022.

Año	Episodios de IRA^a^	Episodios confirmados de virus respiratorios							
		VR (+)	VSR^b^	IA^b^	PIV-3^b^	hMPV^b^	PIV-1^b^	IB^b^	PIV-2^b^
	n (%)	n (%)	n (%)	n (%)	n (%)	n (%)	n (%)	n (%)	n (%)
2019	308 (17,7)	60 (19,5)	40 (66,7)	12 (20,0)	2 (3,3)	3 (5,0)	1 (1,7)	2 (3,3)	0 (0,0)
2020	232 (13,3)	8 (3,4)	6 (75,0)	0 (0,0)	0 (0,0)	1 (12,5)	1 (12,5)	0 (0,0)	0 (0,0)
2021	603 (34,7)	51 (8,5)	48 (94,1)	0 (0,0)	1 (2,0)	3 (5,9)	0 (0,0)	0 (0,0)	0 (0,0)
2022^c^	597 (34,3)	131 (21,9)	102 (77,9)	11 (8,4)	11 (8,4)	6 (4,6)	1 (0,8)	0 (0,0)	0 (0,0)
Total	1740 (100,0)	250 (14,4)	196 (78,0)	23 (9,2)	14 (5,6)	13 (5,2)	3 (1,2)	2 (0,8)	0 (0,0)

IRA: infección respiratoria aguda; VR: virus respiratorio; VSR: virus sincitial respiratorio; IA: influenza A; PIV: parainfluenza virus; hMPV: metapneumovirus; IB: influenza B.

a Los porcentajes corresponden al número total de infecciones respiratorias agudas (N=1740).

b Los porcentajes corresponden al total de episodios de virus respiratorios confirmados para el período evaluado.

c Hasta agosto del 2022.

**Figura 1:** A) Frecuencia de episodios de infecciones respiratorias agudas (IRA) confirmadas mediante inmunofluorescencia directa. B) Frecuencia de episodios de IRA de etiología viral confirmadas mediante inmunofluorescencia directa.

VR: virus respiratorio, VSR: virus sincitial respiratorio.

La mayoría de las pruebas (28,1%) correspondió a niños menores de un año. La positividad viral fue menor en los grupos de 5 a 10 años (8,6%) y mayores de 10 años (9,2%) (tabla 2, figura 2). La detección más alta se registró en 2022 (21,9%), durante la tercera ola de COVID-19 (24,5%) y en otoño (20%). La positividad fue menor en 2020 (3,4%), 2021 (8,5%), así como en primavera (3,7%) y verano (9,7%).

**Tabla 2:** Características de los episodios de IRA con un resultado positivo de virus respiratorios en el Instituto Nacional de Salud del Niño-San Borja, enero 2019 a agosto 2022.

Características		Episodios de IRA (n=1740)	VR negativo (n=1490)	VR positivo (n=250)
		n (%)	n (%)	n (%)
Edad en meses, mediana (RIC)		26 (10‒70)	29 (11‒74)	20 (8‒41)
Grupo etario				
	< 1 año	489 (28,1)	400 (81,8)	89 (18,2)
	1 a < 2 años	334 (19,2)	281 (84,1)	53 (15,9)
	2 a < 5 años	420 (24,1)	356 (84,8)	64 (15,2)
	5 años a 10 años	279 (16,0)	255 (91,4)	24 (8,6)
	>10 años	218 (12,6)	198 (90,8)	20 (9,2)
Sexo				
	Femenino	765 (44,0)	647 (84,6)	118 (15,4)
	Masculino	975 (56,0)	843 (86,5)	132 (13,5)
Tipo de atención				
	Hospitalización	1599 (91,9)	1371 (85,7)	228 (14,3)
	Consultorio externo	45 (2,6)	40 (88,9)	5 (11,1)
	Emergencia	96 (5,5)	79 (82,3)	17 (17,7)
Año				
	2019	308 (17,7)	248 (80,5)	60 (19,5)
	2020	232 (13,3)	224 (96,6)	8 (3,4)
	2021	603 (34,7)	552 (91,5)	51 (8,5)
	2022 (hasta agosto)	597 (34,3)	466 (78,1)	131 (21,9)
Estación del año				
	Otoño	640 (36,8)	512 (80,0)	128 (20,0)
	Invierno	589 (33,8)	502 (85,2)	87 (14,8)
	Primavera	242 (13,9)	233 (96,3)	9 (3,7)
	Verano	269 (15,5)	243 (90,3)	26 (9,7)

IRA: infección respiratoria aguda; VR: virus respiratorio, RIC: rango intercuartílico.

**Figura 2:** Etiología de los episodios de infecciones respiratorias virales agudas confirmadas mediante inmunofluorescencia directa según el grupo de edad.

VSR: virus sincitial respiratorio; IA o IB: influenza A o influenza B, PIV: virus parainfluenza, hMPV: metaneumovirus.

Entre los casos positivos, el VSR fue el más frecuente (78,0%), pero tuvo un descenso en 2020 (2,4%) (figura 1B). En contraste, en 2022 aumentó la detección de otros virus: IA (4,4%), PIV 3 (4,4%) y hMPV (2,4%) (figura 1B, tabla suplementaria 1). El VSR predominó en menores de un año (83,2%) y de 1 a 2 años (86,8%), con picos en 2021 (94,1%), la segunda ola de COVID-19 (93,9%) y en otoño (88,3%). IA e IB representaron 10,0% de los casos, con mayor frecuencia en niños mayores de 10 años (30,0%), especialmente en 2019 (23,3%) y en verano (38,5%). El PIV (6,8%) se detectó principalmente durante la cuarta ola (43,8%). El hMPV (5,2%) fue más frecuente en primavera (33,3%) (tabla suplementaria 1). Las frecuencias anuales se presentan en las tablas suplementarias 2 y 3.

## DISCUSIÓN

Las tendencias de infección respiratoria viral aguda observadas en este estudio muestran una reducción en la positividad a VR durante la primera y la segunda ola de COVID-19 en el Perú (2020-2021), seguida de un incremento posterior. De forma similar, la frecuencia de VR disminuyó durante la pandemia en China ^4^ y en América Latina durante el invierno y otoño del 2020 (estaciones con brotes prepandémicos) ^12^. Además, este patrón fue consistente con un estudio enfocado específicamente en VSR realizado a partir de un registro pediátrico de 260 establecimientos en Tokio, que no reportó brotes de IRA por VSR en 2020, sin embargo, los autores observaron una elevada frecuencia en 2021 ^13^. Esto se debió probablemente a las medidas de salud pública como el confinamiento y el distanciamiento social, que limitaron la transmisión viral y la socialización infantil ^14^.

En el Perú, una cuarentena nacional estricta probablemente contribuyó a más de 100 días de reducción de la circulación de VR ^15^. No obstante, la menor atención a patologías leves en distintos centros de salud pudo conducir a un subregistro. Por otro lado, la alta frecuencia de VR en 2021-2022 sugiere un posible efecto rebote, subrayando la importancia de mantener y fortalecer la vigilancia epidemiológica de VR.

La menor frecuencia de IRA se presentó en los grupos de mayor edad (5-10 años y >10 años), los años 2020 y 2021, y las estaciones de primavera y verano. Estos hallazgos coinciden con estudios de China ^(^^4^, y Alemania ^16^^)^ que mostraron menor incidencia de IRA en niños mayores y durante la fase temprana de la pandemia. Es importante destacar que no se registraron casos de IA o IB en 2020-2021, con un repunte en 2022, tendencia también observada en India ^17^.

Los patrones estacionales coincidieron con hallazgos de Sudáfrica ^18^ y Argentina ^19^. En el Perú, un estudio encontró que el VSR alcanzó sus picos de circulación durante el otoño y el invierno de 2017 y 2018 ^6^, lo que concuerda con nuestros resultados. El resurgimiento de casos en 2021 y 2022 puede atribuirse, en parte, al reforzamiento de la vigilancia epidemiológica.

Las intervenciones no farmacológicas, como el uso de mascarillas, la higiene de manos, el distanciamiento social y la cuarentena, probablemente redujeron la transmisión de VR durante la pandemia ^14^. Si bien estas medidas resultaron beneficiosas, la interrupción de la circulación viral en 2020 puede traer consecuencias ya que usualmente el 90% de los niños experimenta al menos una IRA viral en los dos primeros años de vida ^20^, lo que contribuye al desarrollo de su inmunidad. La menor exposición durante la pandemia pudo dejar a los niños más susceptibles a futuras infecciones, lo que podría traducirse en mayor morbilidad y mortalidad respiratoria en los próximos años. Estos hallazgos resaltan la necesidad de mejorar las estrategias diagnósticas y preventivas mediante el fortalecimiento de la vigilancia epidemiológica en los establecimientos de salud. El incremento de casos de VR en 2022 sugiere la posibilidad de futuros brotes, enfatizando la importancia de la preparación y la vigilancia en el Perú para futuros escenarios similares.

Entre las limitaciones se debe mencionar que, este estudio se realizó en un hospital de referencia, lo que puede restringir la generalización de los hallazgos a otros niveles de atención. Además, podrían haberse omitido casos atendidos en otros centros de atención y de episodios que no buscaron atención. Adicionalmente, la menor frecuencia de IRA en 2020 puede explicarse parcialmente por la menor asistencia a los servicios de salud debido al temor al COVID-19. Asimismo, las diferencias en la estrategia de captación entre 2019-2021 (no protocolizada) y 2022 (vigilancia activa de VR protocolizada) podrían haber influido en la frecuencia de episodios y en la positividad viral observada, limitando la comparabilidad entre periodos, aunque las frecuencias de VR durante el 2019 se mantuvieron similares al 2022. Por otro lado, los datos de 2022 corresponden únicamente hasta agosto, lo que podría limitar la comparación con años previos, no obstante, ya se observaba una positividad viral superior a la registrada durante los primeros años de pandemia. Finalmente, no se puede establecer causalidad por el diseño del estudio. Sin embargo, el estudio presenta la fortaleza de haberse enfocado en casos de IRA confirmados virológicamente mediante pruebas de IFD. Además, proporcionamos análisis detallados por tipo de virus, grupo etario, estación y año, abarcando las tendencias hasta agosto de 2022.

En conclusión, el VSR fue la principal causa viral de IRA, especialmente en menores de cinco años, mientras que la influenza predominó en los grupos de mayor edad atendidos en el INSN-SB. La frecuencia de IRA fue menor a mayor edad, durante los dos primeros años de la pandemia de COVID-19 (2020 y 2021) y durante las estaciones de primavera y verano. La mayor frecuencia de VR observada en 2022 sugiere la necesidad de mantener y reforzar la vigilancia y la preparación ante brotes en el Perú para afrontar futuros desafíos de salud pública relacionados con las infecciones respiratorias.
